# Perspectives on Thermoelectric Energy Conversion in Ion-Exchange Membranes

**DOI:** 10.3390/e20120905

**Published:** 2018-11-26

**Authors:** V. María Barragán, Kim R. Kristiansen, Signe Kjelstrup

**Affiliations:** 1 Department of Structure of Matter, Thermal Physics and Electronics; Complutense University of Madrid, 28040 Madrid, Spain; 2 PoreLab, Department of Chemistry, Norwegian University of Science and Technology, N-7491 Trondheim, Norway

**Keywords:** ion-exchange membrane, thermoelectric power, transported entropy

## Abstract

By thermoelectric power generation we mean the creation of electrical power directly from a temperature gradient. Semiconductors have been mainly used for this purpose, but these imply the use of rare and expensive materials. We show in this review that ion-exchange membranes may be interesting alternatives for thermoelectric energy conversion, giving Seebeck coefficients around 1 mV/K. Laboratory cells with Ag|AgCl electrodes can be used to find the transported entropies of the ions in the membrane without making assumptions. Non-equilibrium thermodynamics can be used to compute the Seebeck coefficient of this and other cells, in particular the popular cell with calomel electrodes. We review experimental results in the literature on cells with ion-exchange membranes, document the relatively large Seebeck coefficient, and explain with the help of theory its variation with electrode materials and electrolyte concentration and composition. The impact of the membrane heterogeneity and water content on the transported entropies is documented, and it is concluded that this and other properties should be further investigated, to better understand how all transport properties can serve the purpose of thermoelectric energy conversion.

## 1. Introduction

Thermoelectricity involves the direct coupling of fluxes of heat and electric charge. The coupling can refer to the way a temperature difference can produce electricity, or to the reverse, how an electric current can create a temperature difference. The first part of the thermoelectric effect, the conversion of heat to electricity, was discovered in 1821 by Thomas Seebeck [1]. The second effect was explored in more detail by Jean Peltier, and is referred to as the Peltier effect. They are linked by the Onsager reciprocal relations (see below). Thermoelectric devices provide the only direct possibility to convert low-temperature heat sources into electric power. This property makes them potential candidates for industrial waste heat conversion. In contrast to heat engines, thermoelectric generators have no moving parts. Thermoelectric energy converters have in practice, since long been made from semiconductors [2], and there are several ideas for their improvement, e.g., nanostructured materials [3,4,5]. The ideas were proposed by Hicks and Dresselhaus already in 1993 [6,7]. Semiconductors are, however, often expensive or rare, and do not offer particularly large Seebeck coefficients, typically 200–300 μV/K, even if colossal values have been found for peculiar conditions [8].

It may therefore be interesting to examine the potential of other conductors. Materials such as solid state ionic conductors and electrolytes [9], or ionic liquids [10,11], are relevant. Granular porous media [12] and electrically conductive polymers [13] also show thermoelectric effects. This review will, however, focus on a large class of materials which may be more accessible, namely ion-exchange materials. In the last several years, the possibility of using ion-exchange membranes in renewable energy technology is in the crosshairs [14,15,16,17,18,19].

The aim of this work is to review state-of-the-art knowledge on thermoelectric energy conversion in cells with ion-exchange membranes. The hope is to provide a basis for further explorations of their use, i.e., in reverse electrodialysis (RED) [18,20]. In a RED concentration cell, isothermal alternating compartments of sea water and brackish water separated by ion-selective membranes permit the production of electric power [21]. Recent work demonstrates that a thermoelectric potential can be added to the RED concentration cell to increase the electromotive force by 10% per 20 K difference for given electrolyte conditions [20]. The class of ion exchange materials may provide cheaper cell components than presently used and help make renewable technologies more competitive. We shall review experimental results from cells with ion-conducting membranes.

The energy conversion that takes place in these cells can be well described by non-equilibrium thermodynamics. This theory relates properties that are critical for energy conversion, and we shall decompose measurements as far as possible in terms of these properties.

The typical experimental cell for thermoelectric energy conversion, or thermocell for short, which is relevant for waste heat exploitation (below 100 °C), can be schematically written as

*T*, Ag|AgCl | MCl(aq) |membrane| MCl(aq) | Ag| AgCl, *T* + Δ*T*

Salt solutions of MCl are separated by an ion-exchange membrane. The electrodes here are reversible to the chloride ion. Other electrodes are also relevant. The temperature difference is ideally across the membrane alone.

The study of such cells is not new. Already in 1956, Tyrrell [22] described this type of cells. In 1957, Hill et al. [23] described the potential difference in terms of irreversible thermodynamics. A year later, Ikeda and coworkers [24,25] reported a thermal membrane potential of 24.6 μV/K with 0.1 M KCl solutions for collodion membranes. However, Tyrrell et al. [22] had obtained a value 10 times higher with ion-exchangers.

Non-isothermal transport phenomena in charged membranes have been reported occasionally in the literature since then [26,27,28,29]. In the 1970s and 1980s, Tasaka and co-workers were central [30,31,32,33,34,35,36,37], while new researchers joined in the 1990s [38,39,40,41,42,43,44,45,46,47,48,49,50,51,52]. Different aspects were studied, such as the influence of the membrane type [40,41,42,51,52], the concentration and nature of the electrolyte solutions in contact with the membrane [39,42], and the membrane-transported entropy of ions [42,45]. Experimental techniques were refined, to resolve thermal polarization of membranes and quantify contributions from thermo-osmotic processes [48]. Temperature effects were also an issue [49].

Non-equilibrium thermodynamics [28,53,54,55,56,57] can be used to describe the conversion of thermal to electric energy. Two sets of variables are then relevant: the practical set according to Katchalsky and Curran [53], which consists of measurable variables, and the set most often used of ionic variables. We consider it an advantage to have two equivalent paths for derivation of expressions to be used in the laboratory, but shall systematically use the practical set and compare it to the other in the end. The practical set is suited to make clear the relation between experiment and theory and give advice on experimental design.

The reader who is interested in the experimental results alone may go directly to the end of Section 4 where we present the final equations, which enable us to explain and compare experimental results.

The main aim of this work is thus to bring out the potential of a new class of materials, the ion-exchange materials, for thermoelectric energy conversion purposes. In spite of good knowledge about thermoelectric generators in general, see, e.g., [12,13], properties of cells with ion-exchange membranes have scarcely been studied systematically. It is our hope that this work can provide a basis and pinpoint needs for further research.

## 2. The Cell

**Figure 1:**
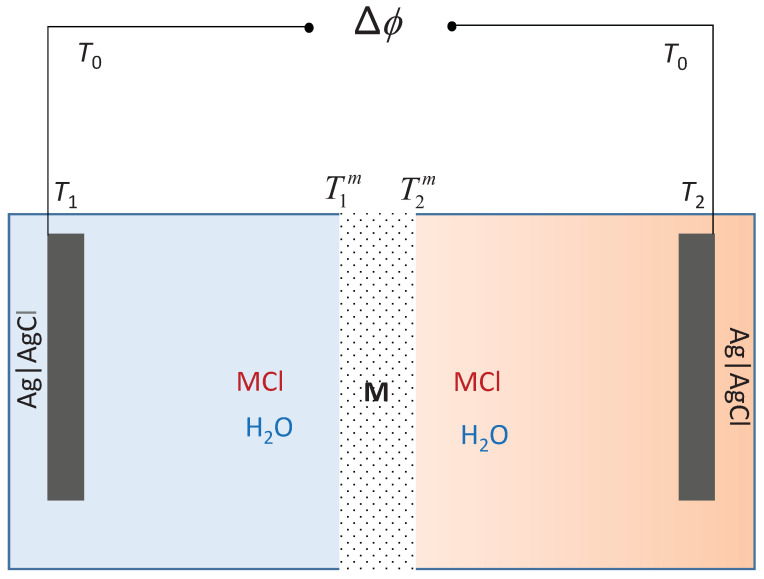
A schematic illustration of the electrochemical cell with electrodes of Ag(s)|AgCl(s), kept at temperatures $T_{1}$ and $T_{2}$. The potentiometer is kept at temperature $T_{0}$. The membrane, M, is surrounded by two MCl electrolyte solutions, of the same or different compositions. $T_{1}^{m}$ and $T_{2}^{m}$ show the temperature at both membrane surfaces.

The typical cell membrane has transport of salt and water, see Figure 1 for an illustration. We consider electrodes of silver and silver chloride, but other electrodes (calomel) have often been used. The membrane is cation- or anion-selective. We can imagine that waste heat sources are used to maintain a temperature difference across the membrane. In a saline power plant, say the reverse electrodialysis plant, there is already a concentration difference across the membrane [21]. A pressure difference can also arise, but this possibility will be neglected for now.

The transport processes that we consider take place along the horizontal axis of the cell. This will be referred to as the *x*-axis. The system is normally stirred and therefore homogeneous in the *y*-*z* plane for any fixed *x*-coordinate. All membrane fluxes can then be given by the scalar *x*-component of the vectorial flux. The membrane surfaces can be assumed to be in *local* equilibrium. We shall mainly examine *emf*-experiments, which are carried out in the limit of very small current densities (open circuit potential measurements). The electrodes are connected to a potentiometer via Cu wires. The potentiometer is at room temperature $T_{0}$, while the electrodes have different temperatures, *T* or T+ΔT, like their thermostated electrolyte solutions, respectively. We are seeking an expression for the *emf* of the cell in terms of properties that can be measured.

The *emf* of the cell in Figure 1, Δϕ, represents the ideal electric work done that can be done by the cell, and can be found by adding contributions along the circuit, from the leads that connect the electrodes to the potentiometer (two contributions giving $\Delta_{\mathrm{ext}}\phi$, from the left and right electrodes (giving $\Delta_{\mathrm{el}}$) and from the membrane ($\Delta_{m}\phi$). The *emf* is thus

(1) $$\Delta \phi =\Delta_{\mathrm{ext}}\phi +\Delta_{\mathrm{el}}\phi +\Delta_{m}\phi .$$

Subscripts on the symbol Δ indicate the origin of the contributions; from the connecting leads (“ext”), from the electrodes a and c (“el”), and from the membrane (“m”). The electrolyte solutions are stirred, so they are isothermal and fully mixed. Therefore, they do not contribute to the *emf*, $\Delta_{\mathrm{aq}}\phi =0$.

## 3. The Electromotive Force of the Ag|AgCl-Cell

We derive the expression for the measured electromotive force (*emf*), Δϕ, of the cell, in terms of measurable properties of the cell. The cell has nine distinct phases—two connecting leads from electrodes to a potentiometer, anode- and cathode-surfaces, two bulk electrolyte solutions, a membrane, and two interfaces between the membrane and the solutions. Out of these nine phases, eight can be considered pairwise physically equivalent, differing only in parameter values. We consider aqueous solutions that are uniform and in local equilibrium with the membrane on each of the membrane sides.

### 3.1. Connecting Leads

The wires connecting the Ag|AgCl electrodes to the potentiometer conduct heat as well as charge. The entropy production per unit volume, $\sigma_{\mathrm{ext}}$, is [55]

(2) $$\sigma_{\mathrm{ext}}=J_{q}^{\prime }\partial_{x}\frac{1}{T}-j\frac{1}{T}\partial_{x}\phi .$$

Here $J_{q}^{\prime }$ is the measurable heat flux, and *j* is the electric current density. The contribution to the overall *emf* is measured under reversible conditions (when $\sigma_{\mathrm{ext}}=0$). The *emf*-contribution depends on the temperature difference as

(3) $${\left(\partial_{x}\phi \right)}_{j=0}=-\frac{1}{T}{\left(\frac{J_{q}^{\prime }}{j}\right)}_{dT=0}\partial_{x}T=\frac{S_{e^{-}}^{*}}{F}\partial_{x}T$$

where we have applied the Onsager relation between transport coefficients, see [56] for details. The definition is in accordance with Haase [58] and with Goupil et al. [59]. The heat transported reversibly with the electric current is the transported entropy of the charge carrier, $S_{e^{-}}^{*}$. For all practical purposes, it is constant with temperature. We integrate Equation (3) for the a-side and the c-side, and obtain the first contribution to Equation (1).

(4) $$\Delta_{\mathrm{ext}}\phi =\frac{S_{e^{-}}^{*}}{F}\left[\left(T_{0}-T-\Delta T\right)+\left(T-T_{0}\right)\right]=-\frac{S_{e^{-}}^{*}}{F}.\Delta T$$

### 3.2. The Electrochemical Reaction at the Interface

The electrochemical reaction takes place at the electrode-solution interfaces. The general expression for the entropy production of the anode contains terms from heat and component fluxes into and out of the electrode surface, see [55] for details. Under reversible conditions only two contributions are effective, the term due to reaction and the electric potential jump:

(5) $$\sigma_{a}=0=-j\frac{\Delta_{a,\mathrm{aq}}\phi }{T}-r^{s}\frac{\Delta_{n}G^{a}}{T}$$

where $r^{s}$ is the chemical reaction rate, and $\Delta_{n}G$ is the reaction Gibbs energy of the neutral components of the chemical reaction. The expression for the right-hand side electrode interface (the cathode) are similar. The reaction rate is proportional to the electric current, $r^{s}=j/F$. The electric potential drop can be alternatively expressed by the electrochemical potential difference of the chloride ion. The *emf* contribution for the anode is therefore

(6) $$\Delta_{a,\mathrm{aq}}\phi =-\frac{\Delta_{n}G^{a}}{F}=-\frac{1}{F}\left[\mu_{\mathrm{AgCl}}\left(T\right)-\mu_{\mathrm{Ag}}\left(T\right)\right].$$

There is a similar contribution for the other electrode, c. Subscript (a,aq) denote that the property belongs to the interface between the solid phases a and the aqueous solution. By adding the two electrode surface contributions, respectively, $\Delta_{a,\mathrm{aq}}\phi$ and $\Delta_{\mathrm{aq},c}\phi$, we obtain the next contribution to the *emf* in Equation (1)

(7) $$\Delta_{\mathrm{el}}\phi =\Delta_{a,\mathrm{aq}}\phi +\Delta_{\mathrm{aq},c}\phi =F^{-1}\left(S_{\mathrm{Ag}}-S_{\mathrm{AgCl}}\right)\Delta T.$$

This expression gives the contribution from the electrode reactions to the Seebeck coefficient.

### 3.3. The Membrane

The *emf*-measurements take place under reversible conditions, and one can safely assume equilibrium at the membrane solution interfaces. This makes it convenient to deal with the membrane as a discrete system [57]. The entropy production has contributions from the membrane transport of heat, mass, and charge. In the membrane frame of reference, we have

(8) $$\sigma_{m}=J_{q}^{{}^{\prime }\left(2\right)}\Delta_{m}\left(\frac{1}{T}\right)-J_{w}\frac{\Delta_{m}\mu_{w}\left(T_{1}\right)}{T_{1}}-J_{\mathrm{MCl}}\frac{\Delta_{m}\mu_{\mathrm{MCl}}\left(T_{1}\right)}{T_{1}}-j\frac{1}{T_{1}}\Delta_{m}\phi .$$

In order to arrive at this expression, we eliminated the measurable heat flux at the 1-side using the constant energy flux through the membrane under steady-state conditions. The choice of variables is not unique, but a practical one. Four flux equations follow. The equation for the *emf* is obtained setting j=0 in the equation for the electric current density. The result is well established, see, e.g., [26,53,56].

(9) $$\Delta_{m}\phi =-\frac{L_{\phi q}}{T_{2}L_{\phi \phi }}\Delta_{m}T-\frac{L_{\phi w}}{L_{\phi \phi }}\Delta_{m}\mu_{w}\left(T_{1}\right)-\frac{L_{\phi e}}{L_{\phi \phi }}\Delta_{m}\mu_{\mathrm{MCl}}\left(T_{1}\right).$$

Concentration differences contribute to the *emf*, not only through the last two terms in Equation (9) but also through the concentration dependence of the entropies. We identify the coefficient ratios by means of Onsager relations:

(10) $$\begin{array}{cc} \frac{L_{\phi e}}{L_{\phi \phi }} & =\frac{L_{e\phi }}{L_{\phi \phi }}=\frac{1}{F}{\left(\frac{J_{\mathrm{MCl}}}{j/F}\right)}_{dT=0,d\mu_{i}=0}=\frac{1}{F}{\left(\frac{J_{M^{+}}}{j/F}\right)}_{d\mu_{i}=0}=:\frac{t_{M^{+}}^{m}}{F}\\ \frac{L_{\phi w}}{L_{\phi \phi }} & =\frac{L_{w\phi }}{L_{\phi \phi }}=\frac{1}{F}{\left(\frac{J_{w}}{j/F}\right)}_{dT=0,d\mu_{i}=0}=:\frac{t_{w}^{m}}{F}. \end{array}$$

With the present choice of electrodes, the transfer of electrolytes follows the cation flux. The transport number $t_{M^{+}}^{m}$ is the fraction of the total charge transfer across the membrane carried by the cation. The water transference coefficient $t_{w}^{m}$ is the average number of moles of water transferred (reversibly) with electric current through the membrane per Faraday of charge. The reversible contribution to the heat flux is given by the entropy flux at isothermal conditions. There are two contributions: the entropies related to the nature of charge transporters and the entropy carried along with the components.

(11) $$J_{q}^{{}^{\prime }\left(2\right)}/T_{2}=J_{s}-S_{\mathrm{MCl},2}^{}J_{\mathrm{MCl}}-S_{w,2}J_{w}.$$

We assume that the reversible contribution to the total entropy flux can be attributed solely to the transport of the ions

(12) $${\left(J_{s}\right)}_{dT=0,d\mu_{i}=0}=S_{M^{+}}^{*}J_{M^{+}}+S_{{\mathrm{Cl}}^{-}}^{*}J_{{\mathrm{Cl}}^{-}}.$$

The relation that can be taken to define the transported entropies $S_{i}^{*}$ of the ions. The last coefficient ratio in the expression for the *emf* can now be identified through its Onsager relation

(13) $$\begin{array}{cc} \frac{L_{\phi q}}{T_{2}L_{\phi \phi }}=\frac{L_{q\phi }}{T_{2}L_{\phi \phi }} & =\frac{1}{FT_{2}}{\left(\frac{J_{q}^{{}^{\prime }\left(2\right)}}{j/F}\right)}_{dT=0,d\mu_{i}=0}\\ & =\frac{1}{F}\left[t_{M^{+}}^{m}S_{M^{+}}^{*m}-t_{{\mathrm{Cl}}^{-}}^{m}S_{{\mathrm{Cl}}^{-}}^{*m}-t_{M^{+}}^{m}S_{\mathrm{MCl},2}-t_{w}^{m}S_{w,2}\right]. \end{array}$$

The *emf*-contribution from the membrane becomes

(14) $$\Delta_{m}\phi =\eta_{S}^{m}\Delta_{m}T-\frac{t_{w}^{m}}{F}\Delta_{m}\mu_{w}\left(T_{1}\right)-\frac{t_{M^{+}}^{m}}{F}\Delta_{m}\mu_{\mathrm{MCl}}\left(T_{1}\right)$$

where we have defined $\eta_{S}^{m}$ as

(15) $$\eta_{S}^{m}:=-\frac{1}{F}\left[t_{M^{+}}^{m}S_{M^{+}}^{*m}-t_{{\mathrm{Cl}}^{-}}^{m}S_{{\mathrm{Cl}}^{-}}^{*m}-t_{M^{+}}^{m}S_{\mathrm{MCl},2}-t_{w}^{m}S_{w,2}\right].$$

The entropy of the electrolyte is the standard entropy minus a term that depends on the logarithm of the activity

(16) $$S_{\mathrm{MCl}}=S_{\mathrm{MCl}}^{o}-2R\ln a_{\pm }.$$

The expression for the membrane potential depends thus on the electrolyte activity via the electrolyte entropy, but also via the last terms in Equation (14). We distinguish now between two well defined experimental situations.

In the first situation, the electrolyte solutions are stirred, and the last terms are zero. In the second case, the temperature gradient across the membrane leads to separation of components across the membrane. At the time this has occurred (t=∞, $J_{w}=0,\;J_{\mathrm{MCl}}=0,\;j=0$), there is a balance of forces. This balance is called the Soret equilibrium. We may neglect coupling between the mass fluxes and find the balance of forces expressed by

(17) $$\Delta \mu_{i,T}=-\frac{q_{i}^{*}}{T}\Delta T\;i=\mathrm{MCl},\;w.$$

The electrolyte contribution (the two last terms of Equation (14)) can be determined in this case. The electrolyte contribution can also be contracted [60] using the Gibbs–Duhem relation for the electrolyte solution on the integrated form, provided the concentration difference is small:

(18) $$\Delta_{m}\mu_{w,T}=-mM_{w}\Delta_{m}\mu_{\mathrm{MCl},T}$$

with *m* as the molality and $M_{w}$ as the molar mass of water. By using Equation (18) to eliminate the chemical potential of water, we have

(19) $$-\frac{t_{w}^{m}}{F}\Delta_{m}\mu_{w}\left(T_{1}\right)-\frac{t_{M^{+}}^{m}}{F}\Delta_{m}\mu_{\mathrm{MCl}}\left(T_{1}\right)=-\frac{2R}{F}\left(t_{M^{+}}^{m}-mM_{w}t_{w}^{m}\right)\Delta_{m}\ln a_{\pm }.$$

The bracketed combination is referred to in the literature as the apparent transport number of the membrane. The chemical potential differences (taken at constant temperature) between the two sides are most often zero in thermocell measurements. They are *not* zero, in the Soret equilibrium state, but this state is difficult to realize in the experiment with membranes, because of the time taken to obtain the state.

### 3.4. The Seebeck Coefficient of the Complete Cell

We consider here the experimental situation with identical electrolytes on the two sides of the membrane. The total cell *emf* is obtained by adding the contributions derived above.

At initial time (t=0, no Soret equilibrium), the Seebeck coefficient $\eta_{S}$ of the cell is

(20) $$\eta_{S}=\eta_{S}^{\mathrm{el}}+\eta_{S}^{m}$$

where

(21) $$\eta_{S}^{\mathrm{el}}=F^{-1}\left(S_{\mathrm{Ag}}-S_{\mathrm{AgCl}}-S_{e^{-}}^{*}\right)$$

and

(22) $$\eta_{S,t=0}^{m}:=-F^{-1}\left[t_{M^{+}}^{m}S_{M^{+}}^{*m}-t_{{\mathrm{Cl}}^{-}}^{m}S_{{\mathrm{Cl}}^{-}}^{*m}-t_{M^{+}}^{m}\left(S_{\mathrm{MCl}}^{o}-2R\ln a_{\pm }\right)-t_{w}^{m}S_{w,2}\right].$$

We combined Equations (15) and (16) in the last step. These relations are suited to interpret experiments. We obtain a clear separation between electrode contribution, solution dependent contributions, and membrane-dependent contributions.

## 4. The *emf* of the Cell with Calomel Electrodes

Most experiments reported in the literature have been done with calomel electrodes, rather than with Ag|AgCl electrodes. We need the expression for the *emf* to interpret these experiments. The cell has Hg|Hg${}_{2}$Cl${}_{2}$ electrodes which are kept at the temperature of the potentiometer, $T_{0}$. A salt bridge with saturated KCl is linking the electrode chambers to the electrolyte solution. The salt bridge on the left-hand side of the cell is exposed to a temperature difference $T-T_{0}$, while the salt bridge on the right-hand side is exposed to a temperature difference $T+\Delta T-T_{0}$. The membrane compartments are kept at temperatures *T* and T+ΔT as before. Therefore, there is no net contribution from the two electrode reactions. There is also no net contribution to the chemical potential differences at t=0. As the two electrodes are symmetrically positioned, these contributions cancel. Furthermore, the expression for the membrane contribution has the same form as for the Ag|AgCl system. When the electrolyte concentrations in membrane compartments are the same, we obtain the membrane contribution $\eta_{S}^{m}$ as defined in Equation (15). The membrane contributions to the *emf* can thus be taken from Section 3.

In addition to the contributions discussed, there is a thermocell contribution from the two liquid junctions. This point has so far been overlooked in the experimental literature.

The entropy production of the salt bridge is

(23) $$\sigma_{lj}=J_{q}^{\prime }\partial_{x}\left(\frac{1}{T}\right)-\frac{J_{\mathrm{KCl}}}{T}\partial_{x}\mu_{\mathrm{KCl},T}-\frac{j}{T}\partial_{x}\phi$$

with the measurable heat flux

(24) $$\frac{J_{q}^{\prime }}{T}=J_{s}-S_{\mathrm{KCl}}J_{\mathrm{KCl}}.$$

With arguments similar to those in Section 3, we find the contribution to the *emf* from the liquid junctions (salt bridges)

(25) $$\begin{array}{cc} {\left(\Delta_{lj}\phi \right)}_{j=0} & =\frac{1}{F}\left[t_{K^{+}}\left(S_{\mathrm{KCl}}-S_{K^{+}}^{*}\right)+t_{{\mathrm{Cl}}^{-}}S_{{\mathrm{Cl}}^{-}}^{*}\right]\left[\left(T-T_{0}\right)+\left(T_{0}-T-\Delta T\right)\right]\\ & =-\frac{1}{F}\left[t_{K^{+}}\left(S_{\mathrm{KCl}}-S_{K^{+}}^{*}\right)+t_{{\mathrm{Cl}}^{-}}S_{{\mathrm{Cl}}^{-}}^{*}\right].\Delta T \end{array}$$

The concentration contributions of the two salt bridges cancel, so that we are left with a thermoelectric contribution only. In the interface region between the salt bridges and the electrolyte solutions of MCl, another *emf*-contribution may arise due to variations in the transport numbers and chemical potentials of the salts MCl and KCl in this region. In general, these variations give rise to a bi-ionic potential that will depend on the cation M${}^{+}$ and the concentrations. For a detailed exposition, we refer to [55]. In the present case, we simply consider M = K and assume that the transport number $t_{K^{+}}=0.5$ throughout the liquid junctions. In this case, the total thermoelectric contribution of the salt bridges to the cell *emf* can be quantified as

(26) $$\Delta_{lj}\phi =-\frac{1}{2F}\left(S_{\mathrm{KCl},2}+S_{{\mathrm{Cl}}^{-}}^{*}-S_{K^{+}}^{*}\right)\Delta T.$$

We expect that this expression provides a good lowest order approximation to the contribution [55]. The observed Seebeck coefficient of a cell with calomel electrodes and KCl electrolyte is

(27) $${\left(\frac{\Delta \phi }{\Delta T}\right)}_{j=0}=-\frac{1}{F}\left[t_{K^{+}}^{m}S_{K^{+}}^{*m}-t_{{\mathrm{Cl}}^{-}}^{m}S_{{\mathrm{Cl}}^{-}}^{*m}-\frac{1}{2}\left(S_{K^{+}}^{*}-S_{{\mathrm{Cl}}^{-}}^{*}\right)-\left(t_{K^{+}}^{m}-\frac{1}{2}\right)S_{\mathrm{KCl},2}-t_{w}^{m}S_{w,2}\right].$$

Whenever the electrolyte is different from KCl, we expect non-trivial, but not necessarily large, deviations from this expression. This complication is avoided when Ag|AgCl electrodes are used, and we therefore recommend these electrodes for measuring thermoelectric potentials.

### Theory of Tasaka and Coworkers

Ikeda [24] made one of the first attempts to derive the thermoelectric potential, but Tasaka et al. [27] were first to use irreversible thermodynamics. They found the following expression for the thermoelectric potential in 1965:

(28) $$-{\left[\frac{\Delta \psi }{\Delta T}\right]}_{j=0}=\frac{R}{F}\left(2t_{+}-1\right)\ln a_{\pm }+\left(t_{+}\alpha_{+}+t_{-}\alpha_{-}\right)$$

with

(29) $$\begin{array}{cc} \alpha_{+} & =\eta -\left(\frac{S_{+}^{o}}{F}+\tau_{0}S_{0}^{o}\right)\\ \alpha_{-} & =\eta +\left(\frac{S_{-}^{o}}{F}-\tau_{0}S_{0}^{o}\right). \end{array}$$

Lakshminarayanaiah [28] derived a similar expression. Not all details were reported, but $a_{\pm }$ is the mean salt activity. The η appearing in Equation (29) was called the differential or pure thermo electric coefficient. In the terminology used here, ($F\eta =t_{M^{+}}^{m}S_{M^{+}}^{*m}-t_{{\mathrm{Cl}}^{-}}^{m}S_{{\mathrm{Cl}}^{-}}^{*m}$). The entropy $S_{i}^{o}$ is the partial molar entropy of an ion, and $S_{0}^{o}$ is the partial molar entropy of the solvent. The superscript *o* refers to the reference-state, infinitely dilute system for all components. The quantity $\tau_{0}$ is the reduced transport number of water ($\tau_{0}=t_{w}^{m}/F$). The transport numbers $t_{\pm }$ are, in our notation, the membrane transport numbers $t_{M^{+}}^{m}$ and $t_{{\mathrm{Cl}}^{-}}^{m}$. In order to see the correspondence, we switch to the notation from the previous section. With this notation, Equation (28) becomes

(30) $$\begin{array}{cc} -F{\left[\frac{\Delta \psi }{\Delta T}\right]}_{j=0} & =R\left(2t_{+}-1\right)\ln a_{\pm }-F\tau_{0}S_{0}^{o}-\left(t_{+}S_{+}^{0}-t_{-}S_{-}^{0}\right)+F\eta \\ & =R\left(2t_{M^{+}}^{m}-1\right)\ln a_{\mathrm{MCl}}-t_{w}^{m}S_{w}^{o}-t_{M^{+}}^{m}S_{\mathrm{MCl}}^{o}+S_{{\mathrm{Cl}}^{-}}^{o}+t_{M^{+}}^{m}S_{M^{+}}^{*m}-t_{{\mathrm{Cl}}^{-}}^{m}S_{{\mathrm{Cl}}^{-}}^{*m}. \end{array}$$

We identified the electrolyte entropy $S_{\mathrm{MCl}}^{o}=S_{M^{+}}^{o}+S_{{\mathrm{Cl}}^{-}}^{o}$. In their derivation, Tasaka and coworkers tacitly made the assumption $\gamma_{+}=\gamma_{-}$, with $\gamma_{i}$ being the ion activity coefficients. This allowed their identification $S_{-}=S_{-}^{o}-R\ln a_{\pm }$. Making also the identification $S_{\mathrm{MCl}}=S_{\mathrm{MCl}}^{o}-2R\ln a_{\mathrm{MCl}}$ and setting the water entropy $S_{w}^{o}\to S_{w}$, we find

(31) $$-F{\left[\frac{\Delta \psi }{\Delta T}\right]}_{j=0}=t_{M^{+}}^{m}\left(S_{M^{+}}^{*m}-S_{\mathrm{MCl}}\right)-t_{{\mathrm{Cl}}^{-}}^{m}S_{{\mathrm{Cl}}^{-}}^{*m}-t_{w}^{m}S_{w}+S_{{\mathrm{Cl}}^{-}},$$

which gives the electrostatic potential difference Δψ across the membrane for a given temperature difference. From invariance of the entropy production, the relation between Δψ and the *emf*-contribution Δϕ between chloride-reversible electrodes is

(32) $$\Delta \phi =\Delta \psi -F^{-1}\Delta \mu_{{\mathrm{Cl}}^{-}}.$$

We identify here $\Delta \mu_{{\mathrm{Cl}}^{-}}=-S_{{\mathrm{Cl}}^{-}}\Delta T$, such that the corresponding *emf*-contribution is given by

(33) $$-F{\left[\frac{\Delta \phi }{\Delta T}\right]}_{j=0}=t_{M^{+}}^{m}\left(S_{M^{+}}^{*m}-S_{\mathrm{MCl}}\right)-t_{{\mathrm{Cl}}^{-}}^{m}S_{{\mathrm{Cl}}^{-}}^{*m}-t_{w}^{m}S_{w}.$$

This is exactly our expression in Equation (15) for the membrane contribution to the cell *emf*, when the electrolyte concentration is uniform. For the KCl electrolyte, the addition of the salt bridge contributions derived in the previous section gives Equation (27). By writing out the activity dependence of the electrolyte entropy, we have

(34) $$-F{\left[\frac{\Delta \phi }{\Delta T}\right]}_{j=0}=t_{K^{+}}^{m}S_{K^{+}}^{*m}-t_{{\mathrm{Cl}}^{-}}^{m}S_{{\mathrm{Cl}}^{-}}^{*m}-\frac{1}{2}\left(S_{K^{+}}^{*}-S_{{\mathrm{Cl}}^{-}}^{*}\right)-\left(t_{K^{+}}^{m}-\frac{1}{2}\right)S_{\mathrm{KCl}}^{o}-t_{w}^{m}S_{w,2}+R\left(2t_{K^{+}}^{m}-1\right)\ln a_{\mathrm{KCl},2}.$$

where the observed activity dependence of the Seebeck coefficient is explained principally by the last term. The term in $2t_{K^{+}}$ is attributed to the membrane. The −1-part, however, is here correctly attributed to the KCl salt bridges. This crucial point has not been pointed out earlier, and is important for interpretation of experimental results.

The electrolyte entropy terms provide here a linear dependence of the Seebeck coefficient on the logarithm of the electrolyte activity. As the electrolyte concentration is varied, the partial molar entropy of water will also change in general. Neglecting its variation with temperature, it can be related to the electrolyte activity by means of the Gibbs–Duhem relation

(35) $$dS_{w}=2RM_{w}md\ln a_{\mathrm{MCl}}$$

with *m* as the electrolyte molality and $M_{w}$ as the molar mass of water. Since the activity and molality are related by $a_{\mathrm{MCl}}=\gamma_{\mathrm{MCl}}m$, the change in $S_{w}$ will generally not be linear in $\ln a_{\mathrm{MCl}}$. For small variations at low concentrations, however, we may approximate

(36) $$S_{w}=S_{w}^{o}+2mRM_{w}\ln a_{\mathrm{MCl}}$$

such that

(37) $$2R\left(t_{K^{+}}^{m}-\frac{1}{2}\right)\ln a_{\mathrm{KCl},2}-t_{w}^{m}S_{w,2}=2R\left(\underset{t_{a}^{m}}{\underset{︸}{t_{K^{+}}^{m}-mM_{w}t_{w}^{m}}}-\frac{1}{2}\right)\ln a_{\mathrm{KCl},2}-t_{w}^{m}S_{w}^{o}$$

where we have again identified what is commonly known as the apparent transport number $t_{a}^{m}$ of the membrane. We see from this approximation that water transport gives a slight change in the slope of the Seebeck coefficient vs. $\ln a_{\mathrm{KCl}}$. While this analysis may be useful, it may also be good to keep the two entropy terms separate, because the activity of water in the electrolyte solutions is generally well known. We will, however, use Equation (37) to interpret the dependence of the observed Seebeck coefficient on the electrolyte activity.

## 5. Review of Experimental Results

In the review of experimental results that follow below, we will first discuss conditions for good experimental results. A compilation of data found in the literature is given in Table A1 and Table A2. We proceed to test the theoretical expressions above using these data. In the end, we address factors that can enhance Seebeck coefficients of thermocells with ion-exchange membranes.

### 5.1. Experimental Issues: Temperature Polarization

The Seebeck coefficient shows that the electric potential measured across an ion-exchange membranes is proportional to the temperature difference between the adjacent electrolyte solutions. Thus, the Seebeck coefficient can be determined from the slope of the plot of the measured potential vs. the temperature difference. Unstirred electrolyte layers next to the membrane surfaces may hamper the determination of the temperature difference across the membrane. If the temperature between the electrolyte solutions is $\Delta T^{\prime }$, the true temperature difference across the membrane is $\Delta T<\Delta T^{\prime }$, and there is a temperature difference $\Delta T^{\prime }-\Delta T$ across the external solution. This situation is called temperature polarization. Using ΔT rather than $\Delta T^{\prime }$ may give a wrong estimate of the Seebeck coefficient [37,48]. Experimental results have shown that thermal membrane potential for cation-exchange membranes increases when solutions are stirred [27,37,48,49]. In accordance with this, Seebeck coefficients for anion-exchange membranes were observed to increase with the solution stirring rate [43,61]. We expect that whether stirring increases or decreases the observed Seebeck coefficient depends on the relative strength of the thermoelectric contributions from the membrane and the external solution. If the membrane has a stronger contribution than the solution, then stirring will increase the Seebeck coefficient of the system, while if the membrane has a weaker contribution than the solution, stirring will decrease the observed Seebeck coefficient.

Temperature polarization can be handled by exploiting its dependence on the electrolyte stirring rate. We assume then that the effect disappears in the limit of infinite stirring. An increase in the stirring rate will decrease the thickness of the polarized layer and increase ΔT across the membrane. Barragán et al. [48] designed a scheme to handle such temperature polarization in a systematic way. Many of the early works did not take this into account [36,37,39]. Moreover, although the solutions next to the membrane surface were stirred, the Seebeck coefficient usually refers to an unknown fixed stirring rate. This makes it difficult to compare results from different authors.

An applied temperature difference may eventually lead to a separation of components. At infinite times, the chemical potential difference will be balanced by the thermal driving force (Soret equilibrium). Jokinen et al. [17] suggested that this effect explains observed discrepancies between experimental reports. The Soret effect can be included in the description, as described by Equation (17). This is not relevant for the short times considered here.

### 5.2. Properties Derived From Experimental Results

#### 5.2.1. Electrode Contributions

We have seen in the theoretical section that there is a distinct contribution to the thermoelectric potential from the entropy of electrode components, when the electrodes are held at different temperatures. These contributions may in certain cases be attractive in the way that they make the cell potential large. Large contributions were for instance obtained from electrode gas reactions [62]. In any case, they are straightforward, understandable contributions arising from thermodynamic entropies.

In the analysis of experiments, the contribution due to simple electrodes, such as Ag|AgCl, can be separated from the membrane contribution. Kjelstrup et al. [47] studied a thermoelectric cell with a membrane stack of 55 Nafion 117 membranes in a cylinder of plexiglass. The Ag|AgCl electrodes were held at different temperatures, and their contribution to the Seebeck coefficient was 0.66 mV/K. This is the same as recently observed [63]. The Seebeck coefficient of a polymer electrolyte membrane Nafion 1110 in a cell with two hydrogen electrodes was equal to 0.67 mV/K [14]. The electrode contribution to this number was small (0.04 mV/K) with 1 bar of hydrogen pressure on both sides. This contribution can change much, however, as the entropy of the gas changes with the logarithm of the hydrogen pressure (see [62]).

When calomel electrodes have been used to measure thermoelectric potentials, the electrode reactions have taken place at the same temperature. Nevertheless, there is a temperature gradient across the liquid junction, different in the two half cells. As we have seen in Equation (30), the electrode components will not contribute to the *emf*, and the concentration variation in the two liquid junctions will not contribute much. The cell symmetry gives a small contribution from variations between the two junctions [55] for KCl solutions. However, the temperature gradient contributions from the two junctions do not cancel when added. The contribution was here computed with good accuracy for a cell with KCl as an electrolyte. With other electrolytes, bi-ionic potentials need to be added [55].

A measurement of the thermoelectric potential of a cell using Ag|AgCl electrodes avoids this uncertainty. These electrodes can also be used for all chlorides, as well as mixtures, and such a measurement is therefore our recommendation for new experiments with such electrolytes.

A novel electrode concept, introduced by Sales et al. [64], may extend the choice of electrodes. Their concept combined an ion-exchange membrane and porous carbon electrodes immersed in aqueous electrolytes. These electrodes permitted the extraction of electric energy from small temperature differences, taking advantage of the created transient temperature gradient. Hu et al. [65] used carbon multiwalled nanotube (MWNT) buckypaper electrodes in a thermo-electrochemical cell, obtaining three times higher efficiencies than in a similar device with Pt electrodes. This suggests that nanostructured electrode materials with fast kinetics and large amounts of electrochemical accessible internal surface area might also significantly enhance the efficiency of a thermoelectric cell with ion-exchange membranes.

Abrahams et al. [66] obtained −1.41 mV/K using Pt electrodes and a 0.4 M aqueous solution of K${}_{3}$Fe(CN)${}_{6}$/K${}_{4}$Fe(CN)${}_{6}$. The electron transfer reaction in this cell involves complexes with high entropy, explaining the large value. Electrolyte complexing agents such as EDTA [67] may similarly produce large effects due to changes in entropy upon complexation.

Electrode reactions which involve gases or complexing agents may enhance the thermoelectric effect [56]. The *emf* is not only proportional to the temperature difference across the cell electrodes; there is also an effect of a pressure difference. This is the so-called streaming potential. The possibility to add the streaming potential to the Seebeck coefficient is interesting, and not yet exploited (see, however, Sandbakk et al. [15]). These authors reported a large enhancement of the isobaric Seebeck coefficient by application of a pressure difference.

#### 5.2.2. Electrolyte Property Variations

As we have seen in the theoretical section, the Seebeck coefficient of a cell with an ion-exchange membrane has a predicted variation with the electrolyte activity via the term $\frac{R}{F}\left(2t_{M^{+}}-1\right)\ln a_{\pm }$ cf. Equation (34). In order to discuss how this proportionality is reflected in published data, we are plotting $\eta_{S}$ vs. $\ln a_{\pm }$. Most data are available for calomel electrodes, so we use Equation (34) for the analysis.

**Figure 2:**
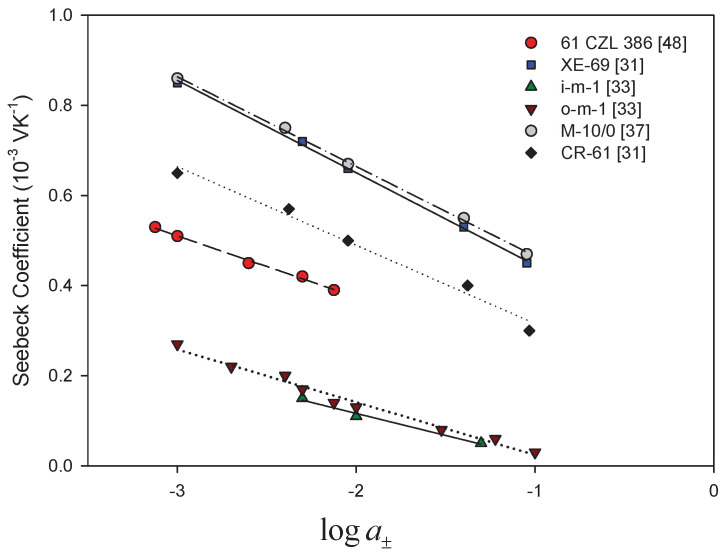
Seebeck coefficients for cells with cation-exchange membranes and a KCl electrolyte of varying concentration, plotted versus the logarithm of the mean salt activity. Calomel electrodes were used in the measurements.

**Figure 3:**
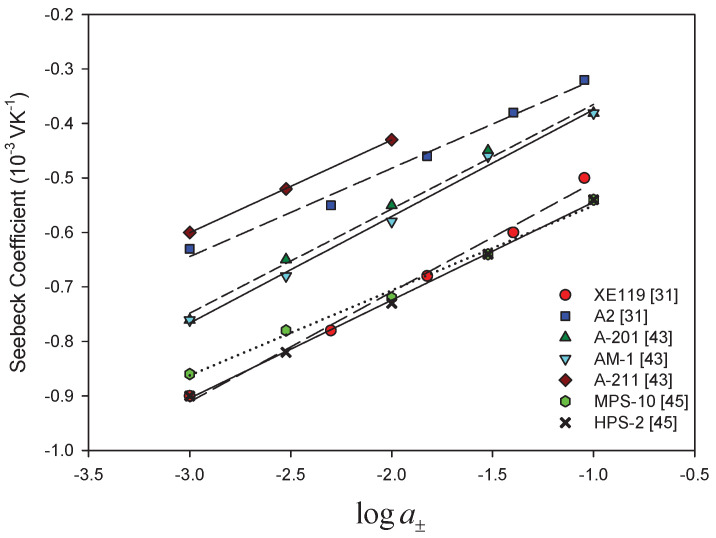
Seebeck coefficients for cells with anion-exchange membranes and a KCl electrolyte of varying concentration, plotted versus the logarithm of the mean salt activity. Calomel electrodes were used in the measurement.

A dependence on $\log_{10}a_{\pm }$ was documented early [26,27,30,31,33,34,35,37,43,45,46,49,52]. Figure 2 and Figure 3 give a summary of results obtained with calomel electrodes and KCl as electrolyte with cation- and anion-exchange membranes, respectively. The observed relation was similar when other electrolytes were used [31,37,43,51], and the slopes were almost the same as the theoretical value 0.1984 mV/K obtained for ideal selective membranes, neglecting the effect of water transport. They were interpreted using the Tasaka expression (Equation (28)).

A deviation from the theoretical ideal value was observed, however, only for increasing electrolyte concentrations for some membrane systems. The deviation was attributed to less selective membranes [30,33,37]. This could be an explanation. However, according to Equation (34), there can also be contributions from water transport and from the liquid junction as well. The entropy of water depends on the electrolyte entropy via Gibbs–Duhems relation (35). It provides a term that is generally not linear in $\ln a_{\pm }$ and provides a change in the linear slope for small concentration variations. Whether this contribution increases or decreases, the value of the slope depends on the sign of $t_{w}^{m}$. The sign gives the direction of water transport in the membrane, which tends to be positive for cation exchange membranes and negative for anion exchange membranes.

By using Equation (37), we estimated a mean value of the apparent transport number for each concentration interval of the curve. For the cation exchange membranes shown in Figure 2, we obtained values between 1 and 0.75. The higher the Seebeck coefficient is, the higher the apparent transport number is. The results of Ikeda et al. [61] also showed an increase of the thermal membrane potential with the apparent transport number in cation-oxidized collodion—and anion aniline-formaldehyde resin-cellophane matrix membranes. They found that the thermoelectric potential increased with the apparent membrane transport number. They did not find the linear trend expected from theory with constant heats of transfer in the membrane. As explanation, they suggested that the heats of the transfer in the membrane were a function of the apparent transport number in the membrane. For the anion exchange membranes shown in Figure 3, we obtained apparent transport numbers that were always smaller than 0.11. They showed less variation. The interpretation of these results is unclear, as long as water transference coefficients and the liquid potential contribution is unknown.

**Figure 4:**
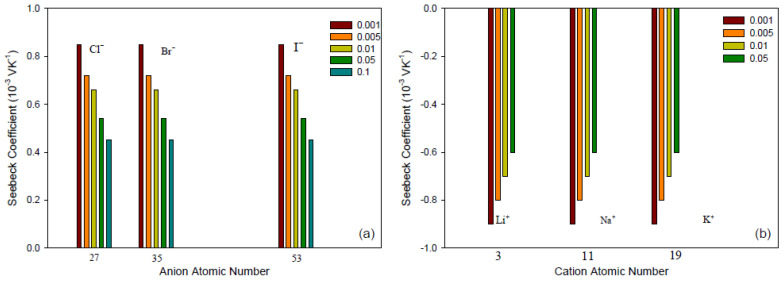
The effect of co-ions on the Seebeck coefficient at different electrolyte molalities [31]. (**a**) Amberlite XE-69 cation-exchange membrane. (**b**) Amberlite XE-119 anion-exchange membrane.

For ideally permselective membranes, we do not expect any effect of co-ions on the thermoelectric potential. This property is seen in Figure 4 for membranes of each type, and for concentrations up to 0.1 M. For these two membranes, the value of the counter-ion transport number estimated from the corresponding slopes in Figure 2 and Figure 3 was practically unity. Similar results about the influence of co-ion were found for all the membranes showing linear behavior of the Seebeck coefficient vs. the logarithm of the mean salt activity. Unfortunately, no results have been found about the effect of co-ions on the thermoelectric potential for the membranes that deviate from ideal behavior. A loss of selectivity of the membrane would involve a larger influence of the co-ion nature at higher electrolyte concentrations, and it would permit analysis if other terms in Equation (34) cause the deviation from the ideal behavior.

We have seen that the sign of the thermoelectric potential of the calomel electrode cell follows mostly from the type of ion-exchange, cation, or anion. This can also be concluded from the term $R\left(1-2t_{M^{+}}^{m}\right)\ln a_{\pm }$. Can the thermoelectric potential of a cell with a particular membrane change its sign? Hanaoka et al. [43,46] suggested this possibility at high electrolyte molalities for some membrane system, for instance, for the Neosepta AM-1 membrane with KIO${}_{3}$ at concentrations higher than 0.1 mol/kg [43]. A sign reversal was observed with the perfluorosulfonic cation-exchange membrane Flemion S [46], for which the thermal membrane potential in HCl solution varied from positive to negative for concentrations above 0.05 mol/kg. They attributed this effect to a water contribution to the thermal membrane potential. This may, however, also be an effect of the bi-ionic potential in the interface region between the salt bridge and the electrolyte solution. The term $R\left(1-2t_{M^{+}}^{m}\right)\ln a_{\pm }$ in Equation (34) may change sign with the transport number and the logarithmic term. The water transference coefficient $t_{w}^{m}$ may also contribute to this behavior through the dependence of the water entropy on the electrolyte activity.

#### 5.2.3. Membrane Structure, Counter Ion, and Water Content

In this review, we examine the contributions to the Seebeck coefficient and how they vary. We have seen in the theoretical section that membrane contributions to the Seebeck coefficient come from the type of membrane (structure), the apparent transport number (the water transference number and the ion transport number), and the transported entropy of the ions in the membrane. Membrane structure and water content may have an impact on all transport properties.

**Figure 5:**
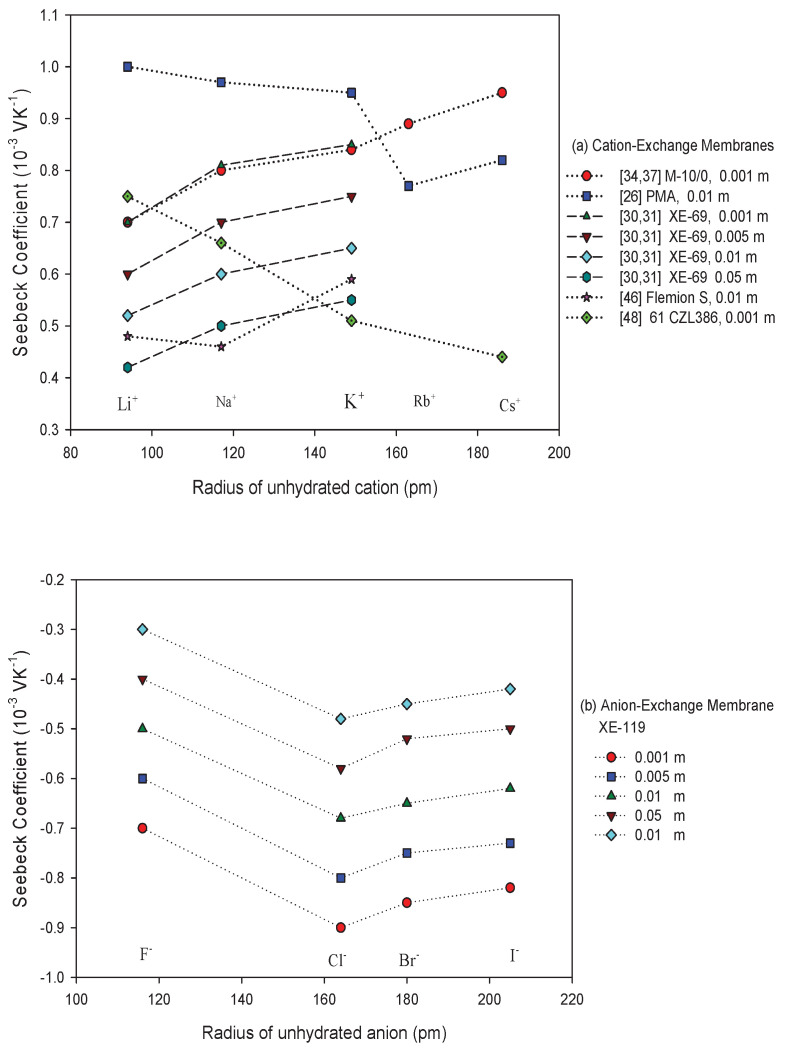
The effect of counter-ions on the Seebeck coefficient for (**a**) different cation-exchange membranes and (**b**) for Amberlite XE-119 anion-exchange membrane [31]. Lines are only a visual guide. Radii values taken from [68].

**Figure 6:**
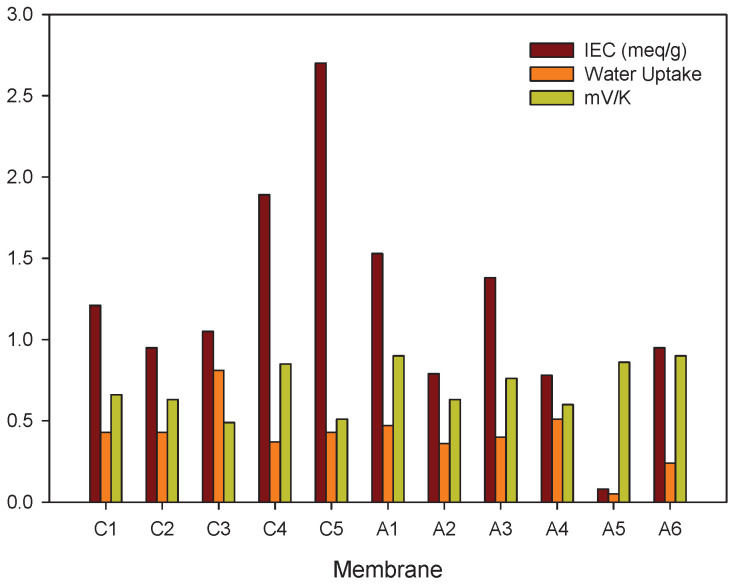
Ion-exchange capacity, water uptake, and Seebeck coefficient (in 0.001 m KCl) for different ion-exchange membranes. Cation-exchange membranes, C1: Nepton CR-61 [37], C2: Nepton CR-51 [37], C3: Interpolymer [37], C4: XE-69 [31], C5: Ionics 61CZL386 [48]. Anion-exchange membranes, A1: XE-119 [37], A2: Nepton AR-111 [37], A3: Aciplex A-201 [43], A4: Aciplex A-211 [43], A5: MPS-10 [46], A6: HPS-2 [46].

- Membrane structure
  Heterogeneous ion-exchange membranes contain more than one charged polymer. It was found that heterogeneous ion-exchange membranes have in general larger thermoelectric potentials than homogeneous ion-exchange membranes, both cationic and anionic membranes [31,37]. Laksminarayanayah [26] and Huda et al. [52] studied the effect of crosslinking of membrane polymers. The degree of crosslinking of the polymethacrylic acid membrane did not have much impact [26] on the Seebeck coefficient. But membranes made of poly(4-vinylpyridineco-styrene) [45] gave thermoelectric potentials that increased with an increase in weight fraction of hydrophobic hydrocarbon matrix, or in the molality of fixed charges. Poly(styrene)-based copolymer anion-exchange membranes with divinylbenzene were investigated in the presence of various electrolyte solutions [52]. Positive contributions were found with KIO${}_{3}$ solutions, and negative for more hydrophobic Cl${}^{-}$-form membranes. The water term seemed predominant for IO${}_{3}^{-}$-form membranes with high water contents. The value of the thermoelectric potential was always negative, which seems to show that the contribution from water on the thermal membrane potential is significant. More systematic studies of the degree of cross-linking as well as membrane hydrophobicity may help obtain better membranes for thermoelectric energy conversion.
- Membrane counter-ion
  The type of ion in the membrane has a clear impact. Figure 5a,b show Seebeck coefficients for different ion-exchange membranes as a function of the radius of unhydrated counter-ion. We see that, in general, the Seebeck coefficient for cationic membranes increases with the atomic number of the cation involved. With HCl and alkali metal chloride solutions, the thermoelectric potential varies roughly with the inverse of the radius of the ions. However, the opposite trend was also observed for some membrane systems [26,46,48]. With anion-exchange membrane Amberlite X-119, the magnitude of Seebeck coefficient increased with increasing molecular weight of halogen ions except for F${}^{-}$ ion. Similar results were found by Huda et al. [52] with poly(styrene)-based copolymer anion-exchange membranes. This property may have some bearing on the transported entropy which enter the expression for $\eta_{S}$.
  Kiyono et al. [44] reported that the size of the Seebeck coefficient depended on the ion-exchange capacity and the membrane water content. They found larger Seebeck coefficients for KCl-solutions than for NaCl, but this was at variance with results of Laksminarayanaiah [28]. It is difficult to see that ion-exchange capacity per se should have an impact on $\eta_{S}$. Indirectly it may play a role, through its impact on the transported entropies, however. No systematic study has been made of the transported entropy and its dependencies.
  At constant selectivity, cation oxidized collodion and anion cellophane membranes gave an absolute value of the thermoelectric potential higher for anion-exchange membranes than for cation-exchange membranes using calomel electrodes [61]. Tasaka et al. [31] measured thermoelectric potentials between 0.85 mV/K and −0.9 mV/K, respectively, with Amberlite XE-69 cation-exchange membranes and Amberlite XE-119 anion-exchange membranes and a 10${}^{-3}$ M aqueous KCl solutions.
- Water content
  It is difficult to separate the effects of water content from that of, say, the ion exchange capacity. The properties are interrelated and the first of them is strongly dependent on the membrane structure. Both properties have only an indirect effect through their impact on the transport numbers and transported entropies appearing in Equation (34). Nevertheless, the larger Seebeck coefficients have been found for membranes with low water content [36,43,45]. Kiyono et al. [45] atributed this behavior to the restricted movement of ions in the membrane with small water content and high weight fraction of hydrophobic backbone. A hydrophobic membrane matrix affects the state of the counter-ion, and therefore also the transported entropy in membrane. Huda et al. [52] altered the hydrophobicity of anion-exchange membranes by changing the length of the alkyl chain of benziltrialkilammonium groups. They observed that the thermoelectric potential decreased roughly with hydrophobicity of the ion-exchange groups.
  In general, the absolute value of thermoelectric potential decreases with the membrane water content (Figure 6). Tasaka et al. [37] also related the decrease in the absolute value of the thermoelectric potential to the water content, leading to a much smaller value of the transported entropy of counter-ion. As can be seen in Figure 6, anion-exchange membranes on the whole have relatively low water content (high apparent transport numbers). It may be explained by the smaller deviation from the theoretical slope of the Seebeck coefficient with the logarithm of the electrolyte concentration observed for anion-exchange membranes (Figure 3). The relatively small ion-exchange capacity does not have much of an impact. The cation-exchange membrane Ionics 61CZL386, with a high ion-exchange capacity, shows a Seebeck coefficient similar to those of other membranes with lower IEC value. In Equation (34), a high water content may mean a high water transport number, positive for cation-exchange membranes [60]. The membrane structure may have an impact on the transported entropy of the counter-ion in the membrane phase.
  When we compare the anion-exchange membranes Nepton AR-111 (homogeneous) and Nepton XE-119 (heterogeneous), we find a higher Seebeck coefficient for the heterogeneous XE-69 membrane with large water content. High water transport numbers are expected for membranes with high water contents. The water transport numbers are negative in anionic membranes [60], so according to Equation (34), a higher water content would increase the Seebeck coefficient. This explanation agrees with the observed experimental results.
  The Seebeck coefficient depends on the solvent, but aqueous solutions seem favorable for a high value [66].

#### 5.2.4. Membrane Temperature: Other Barriers to Transport

The measured potential depended not only on the applied temperature difference but also on the mean temperature of the membrane [49]. For the Ionics 61 CZL 386 cation-exchange membrane and a KCl electrolyte solution, a maximum value was found at 293 K. It was related to the water content of the membrane and to the transported entropies.

Leinov et al. [69] reported thermoelectric coefficients of intact sandstone samples saturated with NaCl brine. With Ag|AgCl electrodes at different temperatures, values were obtained between 0.370 (low salinity, 10${}^{-4}$ M) and 0.055 mV/K (high salinity, 1 M). The sandstone can be seen as a non-selective membrane, which explains the relatively low Seebeck coefficient. It is unclear how it can be used for power generation.

## 6. Competing Thermoelectric Materials

Many materials show thermoelectric effects, and a tailoring to purpose is relevant.

Härtel et al. [70] proposed a novel heat-to-current converter using charged supercapacitors. With commercially available supercapacitors, they observed a coefficient near 0.6 mV/K. Organic materials, in particular intrinsically conducting polymers, can be considered as competitors to classical thermoelectric materials (semiconductors), and their figure of merit has been improved several orders of magnitude in the last few years [71].

Hu et al. [65] showed that carbon-nanotube-based thermo-electrical cells could be used to harvest waste thermal energy. Values of 1.4 mV/K were obtained with carbon MWNT electrodes. Cells with ion-exchange membranes may compete well with these for low temperature waste heat utilization. Xu et al. [72] studied the possibilities of electrolyte solutions confined in nanopore carbon. Using MD simulations, they obtained that the inhomogeneous ion/charge distribution leads to a net electrical potential difference across the solid/liquid interface with energy conversion efficiencies compared with those of conventional thermoelectric materials.

Børset et al. [62] reported the Seebeck coefficient of thermoelectric cells with gas electrodes and a molten electrolyte of lithium carbonate at high temperatures. The presence of an inorganic oxide enhanced the result, increasing it from 0.9 to 1.2 mV/K. Kandhasamy et al. [73] reported larger Seebeck coefficients (1.5 mV/K) for lower temperatures using multi-component mixtures.

## 7. The Potential for Thermoelectric Energy Conversion

For thermoelectric energy conversion, not only the Seebeck coefficient is essential. Other properties need also be considered. To bring out the potential of ion-exchange membranes for thermoelectric energy conversion, we also need to know the thermal and electric conductivity. At a mean temperature $\bar{T}$, the three transport properties can be combined in the lumped variable *N*.

(38) $$N:=\bar{T}\frac{\kappa \eta_{S}^{2}}{\lambda },\;0\leq N\leq 1$$

where

(39) $$\kappa :=-{\left(\frac{j}{\Delta \phi /d_{m}}\right)}_{\Delta T=0}\;\lambda :=-{\left(\frac{J_{q}^{\prime (1)}+J_{q}^{\prime (2)}}{2\Delta T/d_{m}}\right)}_{\Delta \phi =0}$$

are the electrical conductivity at constant temperature and the thermal conductivity of the short-circuited membrane, respectively, and $d_{m}$ is the membrane thickness. The quantity *N* has a lower and an upper bound, stemming from the requirement that the determinant of transport coefficients is positive [55].

The open circuit thermal conductivity, $\lambda_{j=0}$, is related to the conductivity at short-circuit conditions, as follows:

(40) $$\lambda_{j=0}=\lambda \left(1-N\right).$$

The factor ZT is the so-called figure of merit, defined first by Ioffe [74]. It is related to *N* by

(41) $$ZT:=\bar{T}\frac{\kappa \eta_{S}^{2}}{\lambda_{j=0}}=\frac{N}{1-N}.$$

Furthermore, ZT is a non-negative quantity, but now there is no upper bound. When the figure of merit is equal to zero, there is no coupling between heat and charge transfer, and the process is completely irreversible. The other extreme is a completely reversible process, where N→1 and thus ZT→∞. In this situation, the entropy production is (close to) zero; there is no loss of work. In practice, we may seek to maximize *N* or ZT, which means that we want a small thermal conductivity, in order to inhibit irreversible heat transfer, and a large ionic conductivity, in order to reduce ohmic losses. In a dynamic approach, one may choose as objective function for minimization, the total entropy production of the system. It is then possible to also include geometric variables and constraints. This has been done successfully for several unit operations [75].

Ion-exchange membranes are widely used in many applications involving electrochemical energy conversion, such as reverse electrodialysis or fuel cells. The electrochemical process efficiency (voltage efficiency) depends mainly on the overall electric resistance of the device, which is significantly affected by the electrical conductivity of the membrane. The ionic conductivity of ion-exchange membranes has been widely studied in the literature. Temperature, type and concentration of the electrolyte, and membrane water content are parameters that strongly affect the ionic conductivity of the membrane [76,77,78,79,80]. A typical value for ionic conductivity of Nafion membranes at room temperature is 2.3 Sm${}^{-1}$ [77].

Data about thermal conductivity of ion-exchange membranes are, however, scarce. For Nafion membranes values between 0.18 and 0.25 WK${}^{-1}$m${}^{-1}$ have been measured at 20 ${}^{\circ }$C, depending on its water content [81]. Values in the range 0.10 and 0.20 WK${}^{-1}$m${}^{-1}$ were also reported [82] for Nafion membranes, depending on the temperature. The thermal conductivity is found to decrease with increasing temperature [82,83].

An interest in thermoelectric studies has been recently increasing because its also gives direct access to a quantity more difficult to measure, namely the Peltier heat. It is increasingly recognized that local temperature effects and profiles can affect the performance of a fuel cell [84,85]. The temperature profile of such a cell cannot be accurately modeled without knowledge of the (large) Peltier effects [14]. It was probed that thermoelectric contributions to the cell potential can improve the cell performance in the otherwise isothermal reverse electrodialysis cell [20,63].

The importance of the thermoelectric phenomena, their inherent symmetry and impact, lead us to conclude that there are many reasons to increase this area of research and obtain a more complete picture.

## 8. Conclusions and Perspectives

We have seen above that the following can be said about the Seebeck coefficient of thermoelectric cells with ion-exchange membranes:

- It typically varies between absolute values 0.4 and 1 mV/K. Major contributions to this value come from the electrode compartment (including the electrodes) and from water and ion transport in the membrane. These values are relatively large, compared to values for semiconductors. In the calomel electrode system, the sign of the coefficient is typically positive for cation-exchange membranes and negative for anion-exchange membranes. This sign change is attributed mainly to the thermoelectric potential across the salt bridges in the system, but also to water transport when the electrolyte concentration is high.
- It predicts a reduction for ideal membranes with the logarithm of the electrolyte activity. This variation is experimentally validated.
- It has a possible optimal value with respect to the mean temperature across the membrane.
- It does not depend significantly on water content in spite of its dependence on the water transference number.
- It does not depend significantly on the membrane ion exchange capacity. One explanation is that the transport number is the ratio of the ionic and the total conductivity.
- It depends on membrane heterogeneity, probably because this has an impact on the transported entropies of the ions in the membrane.
- It increases in absolute value with the radius of the un-hydrated counter-ion.
- It may have positive contributions from a membrane pressure difference.
- In order to have access to the membrane dependent terms in the Seebeck coefficients, it is advantageous to use electrodes without salt bridges, such as the Ag|AgCl-electrode.

While most of these conclusions are as expected from theory, the ones related to membrane structure and water transport cannot be predicted. They also cannot be given a simple explanation, as they are still not fully understood (see, e.g., [60]). Renewable energy technologies [8,15,18] could benefit from more systematic studies of the impact of the properties on the transported entropies and on the water transference coefficient. The trends in the true Seebeck coefficient that depends on these properties are summarized in Table 1.

Electrode choices and electrolyte conditions, including pressure gradients [15], can help increase the value of the Seebeck coefficient. A temperature difference can lead to a thermo-osmotic pressure. The magnitude as well as the direction of the thermo-osmotic water transfer depends on the membrane [86]. This additional term is little investigated. The understanding of the interplay of thermoelectric and thermo-osmotic phenomena in membrane systems is still lacking and should be pursued.

Not only is the Seebeck coefficient important when it comes to the application of these cells, but the thermal and electric conductivities are as well, as they enter in the figure of merit or the entropy production. Thermal conductivities should be small to help maintain a large temperature difference across the membrane, while electric conductivities should be large to reduce ohmic losses [75,87]. It is a challenge to find ion-exchange membranes that answer to all demands. Finding these is, however, of importance for a large number of interesting waste heat utilization processes.

## Figures and Tables

**Table 1:** Parameters that can increase the Seebeck coefficient of cells with ion-exchange membranes.

Increasing Parameter	Trend	Section	Conclusion, cf. Bullet Point No
Electrolyte concentration	decreasing, logarithmic dependence	5.2.2	1, 2
Counter-ion un-hydrated radius	increasing	5.2.2	7
Membrane hydrophobicity	decreasing	5.2.3	6
Mean temperature	possible optimum	5.2.4	3
Pressure difference	linear increase	5.2.1	8

## Appendix A

**Table A1:** Seebeck coefficient, (Δϕ/ΔT), for cation-exchange membranes.

Membrane (Reference)	Type	Electrolyte		Seebeck Coefficient ($10^{-3}\;{\mathrm{VK}}^{-1}$)	Electrodes
**Collodion** Ikeda [24]		KCl	$1.0\times 10^{-1}$ M	0.052	Ag|AgCl
**Oxidized collodium** Ikeda [61]	Apparent transport number 0.9	KCl	$1.0\times 10^{-1}$ M	0.03	Calomel
**PMA** Laksminarayanaiah [26]	Crosslinked polymetrarylic acid	LiCl	$1.0\times 10^{-2}$ M	1.00	Ag|AgCl
		NaCl		0.97	
		KCl		0.95	
		RbCl		0.77	
		CsCl		0.82	
**Amberlite XE-69-polyvinyl chloride 40%** Tasaka et al. [30,31]	Heterogeneous polystyrenesulfonate	LiCl	$1.0\times 10^{-3}$ M	0.70	Calomel
			$5.0\times 10^{-3}$ M	0.60	
			$1.0\times 10^{-2}$ M	0.52	
			$5.0\times 10^{-2}$ M	0.42	
		NaCl	$1.0\times 10^{-3}$ M	0.81	
			$5.0\times 10^{-3}$ M	0.70	
			$1.0\times 10^{-2}$ M	0.60	
			$5.0\times 10^{-2}$ M	0.50	
		KCl	$1.0\times 10^{-3}$ M	0.85	
			$5.0\times 10^{-3}$ M	0.72	
			$1.0\times 10^{-2}$ M	0.66	
			$5.0\times 10^{-2}$ M	0.54	
		KBr	$1.0\times 10^{-3}$ M	0.83	
			$5.0\times 10^{-3}$ M	0.75	
			$1.0\times 10^{-2}$ M	0.65	
			$5.0\times 10^{-2}$ M	0.50	
		KI	$1.0\times 10^{-3}$ M	0.83	
			$5.0\times 10^{-3}$ M	0.75	
			$1.0\times 10^{-2}$ M	0.65	
			$5.0\times 10^{-2}$ M	0.55	
**o-m-1** Tasaka et al. [30,33]	Oxidized Collodion	KCl	$1.0\times 10^{-3}$ M	0.27	Calomel
			$5.0\times 10^{-3}$ M	0.17	
			$1.0\times 10^{-2}$ M	0.13	
			$5.0\times 10^{-2}$ M	0.07	
**i-m-1** Tasaka et al. [33]	Collodion-sulfonated Polystyrene interpolymer	KCl	$5.0\times 10^{-3}$ M	0.15	Calomel
			$1.0\times 10^{-2}$ M	0.11	
			$5.0\times 10^{-2}$ M	0.05	
**M-10/0** Tasaka [34,37]	Heterogeneous Sulfonate polystyrene+ PVC (40%)	LiCl	$1.0\times 10^{-3}$ M	0.70	Calomel
		NaCl		0.80	
		KCl		0.84	
		RbCl		0.89	
		CsCl		0.95	
		NH${}_{4}$Cl		0.93	
**Nepton CR-61** Tasaka [37]	Homogeneous Sulfonate polystyrene	KCl	$1.0\times 10^{-3}$ M	0.66	Calomel
**Nepton CR-51** Tasaka [37]	Homogeneous Sulfonated phenol resin	KCl	$1.0\times 10^{-3}$ M	0.63	Calomel
**Interpolymer** Tasaka [37]	Sulfonated polystyrene + dried collodion (95%)	KCl	$1.0\times 10^{-3}$ M	0.49	Calomel
**Flemion S** Hanaoka et al. [46]	Porous perfluorosulfonic acid-type	HCl	$1.0\times 10^{-2}$ M	0.053	Calomel
		LiCl		0.48	
		NaCl		0.46	
		KCl		0.59	
		NH${}_{4}$Cl		0.62	
**Nafion 117** Kjelstrup et al. [47]	Homogeneous Sulfonated	HCl	$1.0\times 10^{-2}$ M	0.66	Ag|AgCl
**Ionics 61 CZL 386** Barragán et al. [48]	Heterogeneous sulfonated	KCl	$7.5\times 10^{-4}$ M	0.53	Calomel
			$1.0\times 10^{-3}$ M	0.51	
			$2.5\times 10^{-3}$ M	0.45	
			$5.0\times 10^{-3}$ M	0.42	
			$7.5\times 10^{-1}$ M	0.39	
		LiCl	$1.0\times 10^{-3}$ M	0.75	
		NaCl		0.66	
		KCl		0.51	
		CsCl		0.44	
**Nafion 1110** Kjelstrup et al. [14]	Homogeneous Sulfonated	HCl		0.67	Hydrogen

**Table A2:** Seebeck coefficient, (Δϕ/ΔT), for anion-exchange membranes.

Membrane (Reference)	Type	Electrolyte		Seebeck Coefficient ($10^{-3}\;{\mathrm{VK}}^{-1}$)	Electrodes
**Celophane** Ikeda [61]	Anilline-formaldehyde resin-cellophane matrix	KCl	$1.0\times 10^{-1}$ M	−0.05	calomel
**Amberlite XE-119 polyvinyl chloride 40%** Tasaka et al. [30]	Heterogeneous polystyrene with quaternary ammonium	LiCl	$1.0\times 10^{-3}$ M	−0.9	calomel
			$5.0\times 10^{-3}$ M	−0.8	
			$1.0\times 10^{-2}$ M	−0.7	
			$5.0\times 10^{-2}$ M	−0.6	
		NaCl	$1.0\times 10^{-3}$ M	−0.9	
			$5.0\times 10^{-3}$ M	−0.8	
			$1.0\times 10^{-2}$ M	−0.7	
			$5.0\times 10^{-2}$ M	−0.6	
		KCl	$1.0\times 10^{-3}$ M	−0.9	
			$5.0\times 10^{-3}$ M	−0.8	
		KF	$1.0\times 10^{-3}$ M	−0.7	
			$5.0\times 10^{-3}$ M	−0.6	
			$1.0\times 10^{-2}$ M	−0.5	
			$5.0\times 10^{-2}$ M	−0.40	
		KBr	$1.0\times 10^{-3}$ M	−0.85	
			$5.0\times 10^{-3}$ M	−0.73	
			$1.0\times 10^{-2}$ M	−0.6	
			$5.0\times 10^{-2}$ M	−0.52	
		KI	$1.0\times 10^{-3}$ M	−0.8	
			$5.0\times 10^{-3}$ M	−0.75	
			$1.0\times 10^{-2}$ M	−0.62	
**Aciplex A-201** Hanaoka et al. [43]	Hydrocarbonsulfonic acid	KCl	$1.0\times 10^{-3}$ M	−0.761	calomel
			$3.0\times 10^{-3}$ M	−0.65	
			$1.0\times 10^{-2}$ M	−0.55	
			$3.0\times 10^{-2}$ M	−0.45	
			$1.0\times 10^{-1}$ M	−0.381	
		KIO${}_{3}$	$1.0\times 10^{-3}$ M	−0.64	
			$1.0\times 10^{-1}$ M	−0.36	
**Neosepta AM-1** Hanaoka et al. [43]	Hydrocarbonsulfonic acid	KCl	$1.0\times 10^{-3}$ M	−0.76	calomel
			$3.0\times 10^{-3}$ M	−0.68		
			$1.0\times 10^{-2}$ M	−0.58	
			$3.0\times 10^{-2}$ M	−0.46	
			$1.0\times 10^{-1}$ M	−0.381	
		KIO${}_{3}$	$1.0\times 10^{-3}$ M	−0.551		
			$1.0\times 10^{-1}$ M	−0.168	
**Aciplex A-211** Hanaoka et al. [43]	Hydrocarbonsulfonic acid	KCl	$1.0\times 10^{-3}$ M	−0.6	calomel
			$3.0\times 10^{-3}$ M	−0.5	
			$1.0\times 10^{-2}$ M	−0.4	
**MPS-10** Kiyono et al. [45]	Poly(4-vinylpyridine-co-styrene) with methyl iodide	KCl	$1.0\times 10^{-3}$ M	−0.86	calomel
			$3.0\times 10^{-3}$ M	−0.78	
			$1.0\times 10^{-2}$ M	−0.72	
			$3.0\times 10^{-2}$ M	−0.64	
			$1.0\times 10^{-1}$ M	−0.58	
**HPS-2** Kiyono et al. [45]	Poly(4-vinylpyridine-co-styrene) with 1.6-dibromo-n-hexane	KCl	$1.0\times 10^{-3}$ M	−0.90	calomel
			$3.0\times 10^{-3}$ M	−0.82	
			$1.0\times 10^{-2}$ M	−0.73	
			$3.0\times 10^{-2}$ M	−0.64	
			$1.0\times 10^{-1}$ M	−0.54	
**M-1(4.5)** Huda et al. [52]	Polysterine-based copolymer + 4.5% divinylbencene	KCl	$1.0\times 10^{-2}$ M	−0.592	calomel
		KIO${}_{3}$		−0.485	
**M-1(8)** Huda et al. [52]	Polysterine-based copolymer + 8% divinylbencene	KCl	$1.0\times 10^{-2}$ M	−0.475	calomel
